# Structure guided prediction of Pyrazinamide resistance mutations in pncA

**DOI:** 10.1038/s41598-020-58635-x

**Published:** 2020-02-05

**Authors:** Malancha Karmakar, Carlos H. M. Rodrigues, Kristy Horan, Justin T. Denholm, David B. Ascher

**Affiliations:** 1 Computational Biology and Clinical Informatics, Baker Heart and Diabetes Institute, Melbourne, Victoria Australia; 2 Department of Biochemistry and Molecular Biology, Bio21 Institute, University of Melbourne, Melbourne, Victoria Australia; 3 Victorian Tuberculosis Program, Melbourne Health and Department of Microbiology and Immunology, University of Melbourne, Melbourne, Victoria Australia; 4 Microbiological Diagnostic Unit Public Health Laboratory, University of Melbourne at The Peter Doherty Institute for Infection &Immunity, Melbourne, Victoria Australia; 5 Department of Biochemistry, University of Cambridge, Cambridge, CB2 1GA UK

**Keywords:** Protein analysis, Molecular modelling, Tuberculosis

## Abstract

Pyrazinamide plays an important role in tuberculosis treatment; however, its use is complicated by side-effects and challenges with reliable drug susceptibility testing. Resistance to pyrazinamide is largely driven by mutations in pyrazinamidase (pncA), responsible for drug activation, but genetic heterogeneity has hindered development of a molecular diagnostic test. We proposed to use information on how variants were likely to affect the 3D structure of pncA to identify variants likely to lead to pyrazinamide resistance. We curated 610 pncA mutations with high confidence experimental and clinical information on pyrazinamide susceptibility. The molecular consequences of each mutation on protein stability, conformation, and interactions were computationally assessed using our comprehensive suite of graph-based signature methods, mCSM. The molecular consequences of the variants were used to train a classifier with an accuracy of 80%. Our model was tested against internationally curated clinical datasets, achieving up to 85% accuracy. Screening of 600 Victorian clinical isolates identified a set of previously unreported variants, which our model had a 71% agreement with drug susceptibility testing. Here, we have shown the 3D structure of pncA can be used to accurately identify pyrazinamide resistance mutations. SUSPECT-PZA is freely available at: http://biosig.unimelb.edu.au/suspect_pza/.

## Introduction

Tuberculosis (TB), caused by *Mycobacterium tuberculosis*, is the leading cause of infectious disease death worldwide. In 2017, 10 million people fell ill, and 1.6 million died, from tuberculosis^1^. While a range of antibiotics are available to treat TB, treatment is prolonged, and the increasing emergence of drug-resistant bacteria is a considerable threat to global health. In 2017 alone, an estimated 558,000 people developed multi-drug-resistant tuberculosis (MDR-TB), resistant to the two first-line drugs rifampicin and isoniazid^1^.

Pyrazinamide (PZA) is a first-line drug that exhibits unique sterilizing activity towards both drug-susceptible and MDR-TB^2^. It is responsible for the killing of the persistent tubercle bacilli during the initial intensive phase of chemotherapy, allowing treatment to be shortened from 9 months to 6 months for drug susceptible cases^3^. PZA therapy has been linked to improved outcomes for both non-MDR and MDR-TB, and is being considered as part of the future regimens in combinations with bedaquiline, delamanid, PA-824 and moxifloxacin, which are currently in phase three trials^4,5^.

Despite the highly important role of PZA in clinical outcomes, resistance has largely been underestimated, with up to 20% of non-MDR-TB patients PZA resistant^6^. Being a central drug in current and future regimens, it is important to be able to rapidly and accurately identify resistant isolates and track the emergence and spread of drug resistant strains. *In vitro* drug susceptibility testing (DST) is challenging, expensive and time-consuming as PZA is effective against *M. tuberculosis* only at acidic pH, leading to false resistance rates of up to 70%^7–13^. This has led to the WHO recommending the development of molecular genetics tests.

PZA is a structural analog of nicotinamide and is a pro-drug that needs to be converted into its active form, pyrazinoic acid (POA), by the non-essential enzyme pyrazinamidase, encoded by the *pncA* gene^14,15^. It has been postulated that the mechanism of action of PZA is through POA, which disrupts the bacterial membrane energetics and inhibits the membrane transport function which is necessary for the survival of the bacterium, at an acidic site of infection^16^. PZA resistance has been linked to mutations in a number of genes, including *pncA*, *rpsA*^17^, *panD*^18^, *clpC1*^19^, and the putative efflux pumps *Rv0191*, *Rv3756c*, *Rv3008*, and *Rv1667c*^20^, but mutations in *pncA* are the major mechanism for PZA resistance (70–97%)^21^. While sequencing the *pncA* gene can be a more reliable method to determine resistance than DST, which is prone to missing low-level pyrazinamide resistance caused by non-synonymous mutations in pncA^22^, the development of a genetics based resistance screen is complicated as resistant and non-resistant mutations are found across the entire protein.

To solve the problem of a reliable DST for PZA, we previously showed that protein structural information can be used in a clinical setting to rapidly, accurately and pre-emptively predict drug resistant mutations in *pncA*^23^. This showed that mutations that affected protein folding, flexibility, stability and activity were strongly associated with resistance. Here we have used a comprehensive combination of structure and sequence-based features to develop a predictive tool to characterize novel PncA mutations, which we tested on novel mutations from the Victorian Tuberculosis Program, CRyPTIC^24^ and Miotto *et al*. dataset^25^. This highlights the potential of using structural information to guide the genetic detection of resistance. We have implemented our model through the webserver SUSPECT-PZA (http://biosig.unimelb.edu.au/suspect_pza/), which will enable the rapid structural evaluation of the molecular and phenotypic consequences of any *pncA* nonsynonymous mutation to support informed clinical decisions.

## Results

**Figure 1:**
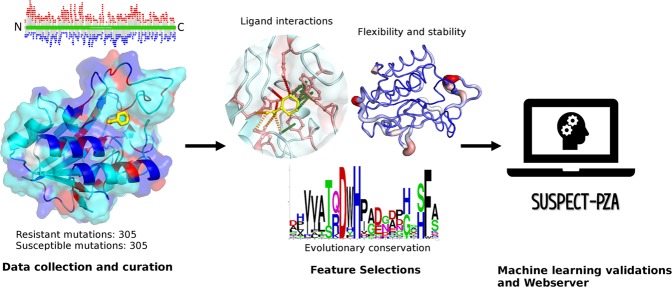
Methodology workflow. The methodology can be divided into three steps. In step 1, data is collected and curated from various tuberculosis databases and articles with experimental evidence like availability of DST results or high-precision laboratory screening study. The curated mutations are shown across both the protein sequence and 3D structure, respectively. The protein sequence and structure of PncA is colored by whether resistant (red) or susceptible (blue) mutations have been observed at that location. Highlighting the difficulty of genomic analysis of pncA, both resistant and susceptible mutations have been observed across many residue positions (cyan). In step 2, effects of mutations on protein stability, dynamics, complementary information regarding the environment characteristics of the wild-type residue (e.g. relative solvent accessibility, residue depth and secondary structure), PZA binding affinity are calculated using different *in-silico* tools. Step 3, all the features are used as evidence to train a supervised machine learning algorithm and after evaluating the performance of the predictive model, the consensus predictions are integrated into a server and can be used to guide clinical resistance detection.

We used a structure-guided approach to understand the structural and functional consequences of variants in the drug target PncA, and machine learning to build an empirical tool that could identify likely resistant mutations. The workflow used to analyze the mutations and train a Random Forest algorithm is shown in Fig. 1 and it comprises three major steps: (1) data curation, which can be subdivided into mutational data set acquisition and protein structure curation; (2) feature analysis, which involves the generation and evaluation of features selected to develop the predictive model to determine novel drug resistance mutations in PncA; (3) machine learning and webserver development, which aims to train, test and validate a supervised machine learning algorithm to accurately predict the susceptibility of the variant followed by a database (SUSPECT-PZA) which has information for all possible variants of PncA.

### Distribution of the mutations on the structure

**Figure 2:**
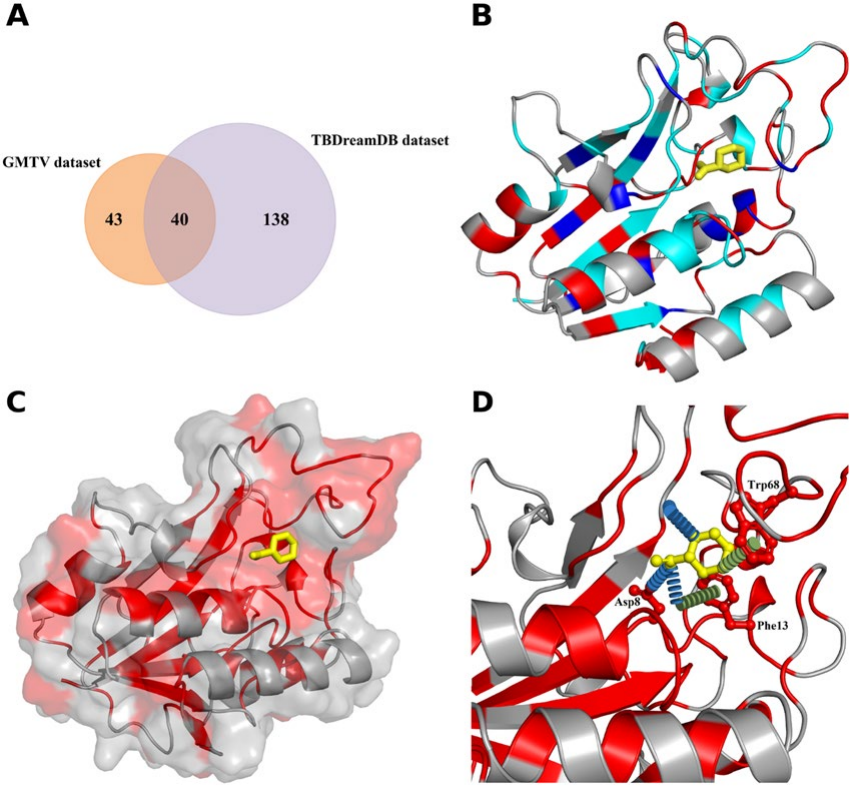
Distribution of clinical resistant and susceptible mutations in PncA. (**A**) Venn diagram representing the distribution of clinical mutations in the different datasets used to build the predictive model. (B) Clinical resistant and susceptible mutations mapped on the crystal structure. Amino acid positions where both susceptible and resistant mutations were seen are colored in cyan and emphasizes the need for a better and improved tool to classify them accurately. (**C**) Surface view of PncA with the docked PZA (yellow, ball and stick representation). Clinical resistant mutations, shown in red, are not just located at the PZA binding site, but are spread equally throughout the whole protein. (**D**) Molecular interactions between PZA (yellow sticks) and the surrounding amino acids which are part of the catalytic triad (Asp8) and substrate binding site (Trp68, Phe13). Hydrogen bonds are shown as blue dashes, and π-interactions as green dashes.

We curated a dataset of 1322 nonsynonymous substitutions with high quality experimentally measured PZA susceptibility (71 susceptible mutations from GMTV^26^, 12 resistant mutations from GMTV^26^, 178 resistant mutations from TBdreamDB^27^, Fig. 2A, 547 resistant and 514 susceptible mutations from experimental saturation mutagenesis^28^). After removal of duplicate mutations, we were left with a dataset of 610 mutations, which included 305 susceptible and 305 resistant mutations. Mapping the complete set of curated 610 nsSNVs (Fig. 1) and just the clinical variants only (Fig. 2B) onto the crystal structure of PncA revealed that variants were distributed throughout the entire protein structure, complicating resistance inference from sequence analysis. We also observed that the resistance mutations were not solely localized at the drug binding site but distributed throughout the protein (Fig. 2C).

PncA is a small protein molecule which constitutes of 186 amino acids. The experimental crystal structure of the drug (PZA) bound to the enzyme (PncA) was unavailable. Therefore, PZA was *ab* initio docked into the experimental crystal structure of the holo-wild-type PncA protein (PDB ID: 3PL1^29^). The docked structure revealed that PZA formed key interactions within the proteins active site, which includes the catalytic triad (Asp8, Lys96, and Cys138), substrate-binding residues (Trp68 and Phe13), and the iron center (Asp49, His51, His57, and Fe 21). Analysis of the molecular interactions with Arpeggio^30^ highlighted a strong network of polar and π- interactions between PZA and PncA (Fig. 2D).

### Structural, biophysical and evolutionary consequences of PncA mutations

**Figure 3:**
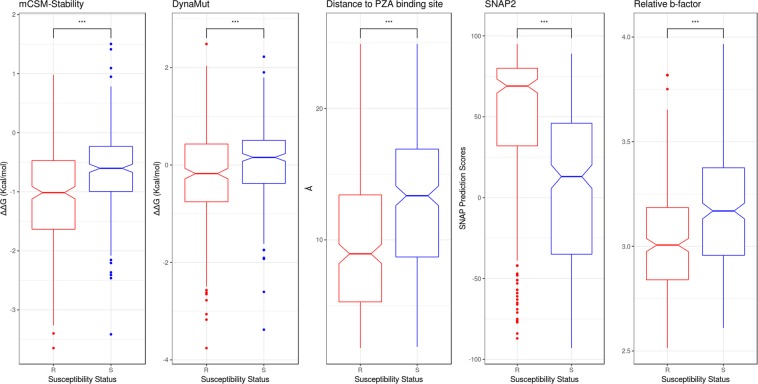
PCA analysis of key molecular features distinguishing resistant and susceptible mutations. Features used for model building are represented as boxplots for explanatory data analysis. The resistant associated mutations (R) are represented as red and the susceptible mutations (S) as blue. (***p < 0.0001, Welch two sample t-test).

Looking at the SNAP2^31^ and PROVEAN^32^ scores, which consider evolutionary information to predict functionally important nonsynonymous mutations, we observed that resistant mutations were always associated with deleterious scores, while susceptible mutations were scored neutral (Table S1; Fig. 3). This suggest that although mutations were spread throughout the protein, mutations associated with resistance were having a stronger effect on the structure and function of the protein.

The wild-type environment also provided information to differentiate between resistant and susceptible mutations, which included relative solvent accessibility (RSA), residue depth and secondary structure of the wild-type residue (Table S1; Fig. 3). This showed that resistant mutations tended to be found at buried residues that were less solvent exposed (average RSA of 0.18 for resistant mutations compared to 0.39 for susceptible; average residue depth of 1.09 Å for resistant mutations compared to 0.75 Å for susceptible; Table S1). These values were consistent with susceptible mutations being in regions that have milder effects on protein stability and activity than the resistance mutations.

The impact of the resistant and susceptible mutations on protein folding, stability and conformation were assessed using biophysical tools which relies on graph-based signatures to calculate the change in Gibb’s free energy, like mCSM-Stability^33^, DUET^34^ and DynaMut^35^. The effect of the mutations on the binding affinity for PZA were assessed using mCSM-Lig^36^. We observed that resistant mutations led to large decreases in PncA stability and conformational flexibility, while susceptible mutations were associated with milder changes (Table S1; Fig. 3). This is consistent with what we have observed previously for non-essential and drug activating proteins^37^. While resistant mutations, however, tended to be located closer to the PZA binding site (average < 10 Å from the PZA; Fig. 3), we did not see a significant difference in the distribution of the effects of resistant and susceptible mutations on PZA binding affinity (Table S1, Fig. S2), likely due to the importance of other molecular effects leading to resistance.

### Machine learning to predict PZA resistance

**Figure 4:**
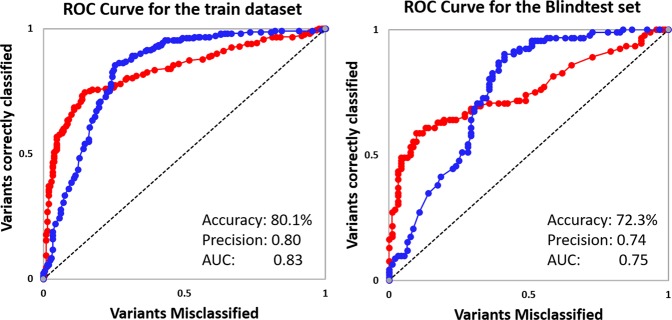
Evaluation Metric for machine learning. Receiver Operating Characteristic (ROC) curves of PZA classifier obtained using the structural and functional consequences of the mutations to accurately identify resistant (red) and susceptible (blue) mutations. (AUC = area under the curve).

Building on this structural and sequence-based analysis, we tested whether the information generated from these features could be used to train a supervised machine learning algorithm capable of accurately predicting resistant mutations in PncA. We grouped our features into five distinct categories: stability, dynamics, evolutionary conservation, ligand interactions and backbone geometry (structural environment). The performance of predictive models trained on each class of feature was evaluated separately to explore the contribution of each class to the predictive model (Table S2; Fig. S2). We were able to confirm that the individual categories of features did not yield a good metric for a reliable predictive model, but in combination using 10-fold cross-validation, models trained using Random Forest algorithm yielded a more balanced and accurate performance, highlighting the synergistic effect of these features. The final model correctly classified 80.1% and 72.3% of mutations in the training and blind datasets, respectively (Fig. 4; Table 1). The comparative performance across iterative non-redundant blind datasets suggested that the model was not overfitted.

**Table 1:** Evaluation metrics across the train and blind test datasets.

	Total nsSNVs	Resistant nsSNVs	correctly classified variants SUSPECT-PZA (%)	Susceptible nsSNVs	correctly classified variants SUSPECT-PZA (%)	PPV (%) (95% CI)	Accuracy (%)
Training dataset (70%)	426	213	159 (74.5)	213	182 (85.5)	83.7 (78.6–87.8)	80.1
Blind test dataset (30%)	184	92	56 (60.8)	92	77 (83.7)	78.9 (69.5–85.9)	72.3
CRyPTIC dataset^24^	355	325	266 (81.8)	30	15 (50.0)	94.7 (92.5–96.2)	79.2
CRyPTIC novel nsSNVs	75	67	67 (74.6)	8	4 (50.0)	92.6 (86.0–96.2)	72.0
Miotto *et al*. dataset^25^	98	92	82 (89.1)	6	2 (33.3)	95.4 (92.1–97.3)	84.8
Miotto novel nsSNVs	44	43	35 (81.4)	1	0	97.2 (96.8–97.6)	79.5
Stellenbosch University and CDC, USA nsSNVs^38^	8	5	3 (60.0)	3	3 (100)	100	75.0
Victorian TB novel nsSNVs	7	4	4 (100)	3	1 (33.3)	66. 7 (47.3–81.7)	71.4

Accuracy = (TP + TN)/(TP + TN + FP + FN); TP: True positives, TN: True Negatives, FP: False Positives, FN: False Negatives

PPV: Positive predictive value, predicting PZA resistance

(nsSNVs - non-synonymous single nucleotide variant).

Analysis of our model revealed that PncA-resistant mutations were associated with large changes in protein folding and stability (mCSM-Stability scores < −0.9 Kcal/mol; p < 0.0001, Welch Two Sample t-test) and conformational flexibility (DynaMut score < 0.78 Kcal/mol; p < 0.0001, Welch Two Sample t-test) or located in close proximity to the catalytic triad and substrate-binding site (<10.8 Å; p < 0.0001, Welch Two Sample t-test). Alternatively, susceptible mutations had a relative b-factor value of ≥3.19 (p < 0.0001, Welch Two Sample t-test), residue depth of ≥0.9 (p < 0.0001, Welch Two Sample t-test), distance from PZA greater than 11.9 Å and mild effects on protein stability (SDM scores ≥ 2.68 Kcal/mol; p < 0.0001, Welch Two Sample t-test).

### Validation using Clinical Datasets

We next validated our model using variants reported in the recently published CRyPTIC dataset^24^. 355 *pncA* nsSNVs associated with PZA resistance were reported, of which 75 were not present in our training dataset. Our model correctly classified 79.2% of the mutations across the whole dataset (355 mutations), and 72.0% of those non-redundant in amino acid position with the training data (75 mutations). The positive predictive value was 94.7% (95% CI [92.5% to 96.2%]).

We also validated our empirical classifier using the dataset reported by Miotto *et al*.^25^, which contained 98 nsSNVs graded by the confidence of their association with phenotypic drug resistance. 44 out of the 98 nsSNVs reported in the paper were not present in our training dataset. We accurately predicted the drug susceptibility of 84.8% of the polymorphism across the whole dataset (98 mutations), with an accuracy of 79.5% for those mutations not included in the training data (44 mutations). The positive predictive value was 95.4% (95% CI [92.1% to 97.3%]). We observed mutations such as Q10P (21 cases reported), W68G (16 cases reported) and I133T (17 cases reported) with 0.98 probability associated with resistant phenotype^22^ and categorized as high confidence for association with resistance, moderate confidence for association with resistance and minimal confidence for association with resistance respectively^25^ were all classified as resistant by our predictive model, highlighting the sensitivity of the prediction.

Mutations reported by Miotto *et al*.^25^ under the “no association with resistance” category, including I31T, L35R and T47A were predicted as resistant, and I6L as susceptible. This is consistent with the available experimental data^24,28^, highlighting the advantage, accuracy and versatility of our approach. A closer look into the different biophysical scores for the resistant associated mutations revealed that they had large predicted destabilizing values for protein conformational flexibility (I31T, −2.49 Kcal/mol) and stability (I31T, −3.46 Kcal/mol) and one was located very close to the catalytic triad (T47A, <6 Å).

Our predictive model was further validated on PZA DST screening at 100 μg/ml of clinical isolates from culture collections at Stellenbosch University, South Africa (865 isolates) and the Centers for Disease Control and Prevention (CDC), Atlanta, USA (185 isolates)^38^. They identified 49 isolates with a susceptible phenotype containing 8 nsSNVs. All nsSNVs with an MIC < 50 μg/ml were correctly classified by our model as susceptible (E37V, D110G, T114M). Whitfield and colleagues suggest that those isolates with an MIC > 50 μg/ml should be considered clinically resistant, of which our model classified three as resistant (A170V, V130A and L35R) and two as susceptible (V163A and V180I). Overall, our model had a 75% agreement with the DST results and a positive predictive value of 100%

### Application within a Clinical Setting

In a prospective genomic sequencing and DST analysis of over 600 Victorian clinical TB isolates, 7 *pncA* variants were detected in 11 variants phenotypically resistant to PZA, none of which were present in our training dataset. Our model correctly classified five out of seven variants as resistant (71.4% accuracy). The remaining two mutations, G108V and Q10H, which were susceptible according to the DST results were predicted to confer resistance and consistent with other experimental findings^24,25,28^. Both variants, had a SNV frequency of <0.5, which is known to impact upon the reliability of the DST results. This highlights the potential clinical power of our model.

Expanding our analysis, four additional *pncA* mutations (S104R, V128G, Y95R and E15A) were identified in Victorian clinical TB isolates lacking DST results. Both S104R and V128G were predicted as resistant by our model, consistent with previously reported DST results^24–28^. The remaining two mutations, Y95R and E15A, have not been reported previously. Our model suggests both mutations to confer susceptibility to PZA.

### SUSPECT-PZA webserver

We have developed a user-friendly, freely available web server SUSPECT-PZA (StrUctural Susceptibility PrEdiCTion on PZA), http://biosig.unimelb.edu.au/suspect_pza/, which is a database for all possible variants of PncA. There are two different input options (Fig. S2): the first one is the “Single Mutation” option which allows the users to input one mutation for analysis. The basic format required by the server for this input option is that the mutation must be specified as a text string containing the wild-type residue one-letter amino acid code, its corresponding position on the structure and the mutant one-letter amino acid code. The second option is the “Mutation List”, which allows the user to upload a list of mutations, in the same specified format as above but in a file for batch processing (Fig. S3). Sample submission entries are available to assist users to submit their mutations for analysis and an additional help page via the top navigation bar.

**Figure 5:**
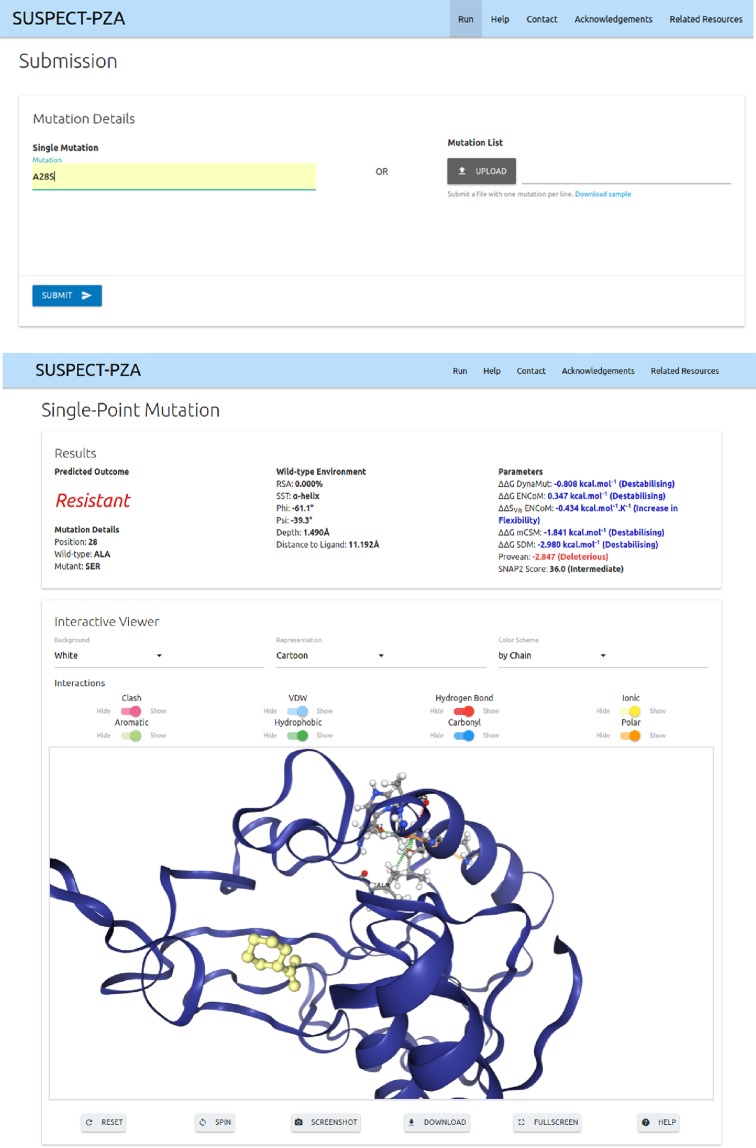
SUSPECT-PZA webserver Single point mutation prediction result page. The predicted outcome of the submitted mutation is displayed along with complimentary information on features used to aid in the development of the tool. The interactive 3D viewer allows user to further analyze non-covalent interactions for both wild type and mutant residues on the protein. A variety of controllers are provided to customize molecule representation.

Figure 5 shows a snapshot of the output page for the “Single Mutation” option. The web server displays the prediction outcome (Resistant / Susceptible) along with details of the user input data, information on the wildtype residue environment and features used for prediction. In addition, there is an interactive 3D viewer, built using NGL^39^, which allows analysis of non-covalent inter-residue interactions for the position specified in the input calculated using Arpeggio^30^ for both wild-type and mutant structures. The results for the “Mutation List” option is summarized in a downloadable table. The users can access details of individual mutation as shown in Fig. S4. There is a 3D viewer at the bottom of the page in which the residues in the input list is colored according to the predicted effect (Fig. S5).

## Discussion

PZA was discovered in 1948 in an *in vivo* screen of nicotinamide derivatives in a structure-activity relationship study^40^ and used as anti-tuberculosis drug in 1952 for the first time. Till the 1970’s PZA was used as a second-line drug to treat TB, until they discovered the sterilizing activity and reduction in treatment duration in combination with isoniazid and rifampicin. There has been a lot of studies conducted since then and with the continued usage of the drug to treat TB, there has been an increased incidence of resistance associated with it. Being an important first-line drug, accurate and rapid evaluation of PZA susceptibility is crucial for successful management of patients with either susceptible or drug-resistant TB. The existing molecular phenotypic tests are considered poorly reliable, expensive, and has a long turnaround time. To account for this situation there is an urgent requirement to develop a rapid, reliable and affordable molecular PZA DST. As resistance mutations are spread all over the length of the PncA protein, it is quite challenging to develop a new method. In this study, we establish a novel computational methodology to better understand the structural and functional consequences of drug resistance mutations by exploiting the protein’s 3D structure. Using supervised machine learning algorithm, we developed an empirical tool to determine novel drug resistance in PncA followed by a database which has information on all possible variants of PncA.

The primary focus of our work is on missense non-synonymous mutations as these typically have more subtle molecular effects that can be harder to predict, than in-frame and frameshift indel mutations that have a much larger deleterious effect on PncA structure and function and are all classed as high-confidence resistant mutations. The structure-based tools implement the concept of graph-based signatures to predict the effect on single point mutations for protein stability. To assess changes in conformational flexibility, graph-based signatures were integrated with normal mode analysis to predict the impact on the protein structure. Scores for these features which were calculated as change in Gibb’s free energy (ΔΔG) provided important molecular information on resistant mutations, signifying larger effects on protein folding and dynamics and minimal effect on PZA binding affinity. Interpreting the results, we observed, resistance mutations were seen to affect protein activity and function through destabilization of the protein structure and conformation. It even helped in correlating earlier findings where resistant isolates were not associated with a loss of bacterial fitness^41^ due to the fact that PncA was involved in nicotinamide recycling pathway rather than in its synthesis. These structural insights have been used to guide clinical decisions for novel PZA mutations^23^.

Phenotypic DST which is the current “gold standard”, which encompasses methods like Wayne and Bactec MGIT 960, suffers from poor reproducibility. Discrepancies among the results lead to considerable doubt over the clinical significance of the method. Next-generation sequencing based diagnostics can be an alternative for innovative tools to reduce false detection of PZA resistance cases and fast and accurate detection of drug resistance by molecular DST^42^. In the past couple of years researchers have used different techniques to come up with a better and consistent methodology to detect and determine resistance in PZA. Stoffels *et al*.^41^ conducted an elaborate study on 14-year complete capture of clinical isolates, where he found frequency of spontaneous acquired resistance to be 10^−5^ bacilli *in vitro*. Miotto *et al*. 2014 work generated the minimum dataset of mutations that should be included in any molecular test for PZA, paving the way for predicting PZA resistance using new genome-based technologies^22^. This was followed by Farhat *et al*. 2016 comprehensive web-based dataset^43^. Though all these approaches were a step up from the existing phenotypic DST, they do not provide information on novel variants. The advantage with our database is it provides information on all possible variants for PncA. This data provides a basis for use as part of any molecular DST, needed for the valid interpretation of data generated by massive sequencing approaches.

Interestingly, comparing performance of SUSPECT-PZA across datasets used to train earlier methods, we observed that the weakest performance was across variants classified as susceptible. However, many of these mutations have been observed in clinically resistant isolates. Our biophysical analysis and SUSPECT-PZA predictions would be consistent with these mutations potentially being misclassified previously.

We also compared our empirical models output to the “revised DST” of Miotto *et al*.^22^, where they accounted for enzymatic activity and structural analysis to adjust for possible errors in phenotypic DST. There were 178 missense mutations listed, of which 162 were labelled resistant (R) and 17 were labelled susceptible (S). Our model predicted 88.9% (144/162) of the resistant mutations and 58.8% (10/17) of the susceptible mutations accurately. The positive predictive value was 95.4% (95% CI [92.1% to 97.3%]). The primary divergence from the Miotto classifications was in predicting susceptible mutations. This is likely due to discrepancies in phenotypic and molecular DST results from different laboratory setups^16^. For example, mutations reported as susceptible in the “revised DST” like L159V, F81S, A102V, T135S, T168I and A46V were unanimously reported as resistant in other studies^24,26–28^. Our predictive tool also predicts them to be resistant and hence, proves to be more reliable, reproducible, free to use and a fast alternative to the existing gold standard methods.

This study highlights the power of using computational prediction of the structural consequences of variants in PncA to identify likely pyrazinamide resistance mutations, a critically important first-line drug in the treatment of tuberculosis. This approach, however, is not limited to pncA and has been developed for application to other antimicrobial agents like bedaquiline^44^, a last line resort to treat multi-drug and extremely drug resistant TB. A major advantage of our tool is that it was built using a very well-balanced dataset. In case of mutations reported as both susceptible and resistant in the same or different datasets, we looked for frequency of occurrence and clinical information. We have extensively evaluated the method through both cross-validation and independent non-redundant blind tests, which provide a measure of a methods applicability and robustness. Across all test sets the method performed equally well, providing strong confidence in the approach. As with all machine learning approaches, the availability of more phenotypic and clinical data will enable the development and validation of stronger approaches. This will be an iterative approach moving forward. The other aspect to improving our predictive model is through the inclusion of new features or parameters. We have shown previously that this approach can even capture strain dependent variations in resistant patterns^23^. While we did not have the data available to build into our current model, we next aim to integrate lineage specific information, which will enable more refined and personalized predictions. This comprehensive web server can be used in clinical settings as an improved diagnostic tool to help realize the power of whole genome sequencing diagnostic approaches.

## Methods

### Data set

A list of 610 nonsynonymous single-nucleotide mutations (nsSNVs) of pncA was obtained from the GMTV (Genome-wide Mycobacterium tuberculosis Variation) Database Project^26^, Tuberculosis Drug Resistance Mutation Database^27^, and saturation mutagenesis^28^. The clinical validation datasets used in the paper were from CRyPTIC^24^ and Miotto *et al*.^25^.

### Modelling the biophysical consequences of missense mutations

We have developed a comprehensive *in silico* mutational analysis platform that uses graph-based signatures to represent the 3D structure of a protein and quantitatively predict the molecular consequences of point mutations on protein structure, function and interactions^30,33–36,45^. This has been used to characterize and preemptively identify likely resistance mutations in drug targets^23,37,46–54^. Using these tools, we assessed the molecular consequences of each mutation on the structure of PncA and drug activation.

The experimental crystal structure of holo-wild-type PncA (PDB ID: 3PL1)^29^ was minimized in Prime, and PZA docked into the active site using Glide (Schrödinger Suite). The effects of mutations on PncA folding and stability were assessed using SDM^55^, mCSM-Stability^33^ and DUET^34^, and their effects on protein flexibility and conformational was predicted using normal mode analysis by DynaMut^35^. The effect of the changes on the binding affinity of PZA towards PncA were predicted using mCSM-Lig^36,56^. These approaches are novel machine-learning algorithms. We also included structural information of the wild-type residue, including relative solvent accessibility, residue depth, secondary structure and dihedral angles of the PncA chain φ (phi) and ψ (psi). Additionally, SNAP2^31^ and PROVEAN^32^ were used to provide additional evolutionary information. Moreover, the scores calculated for the various structural and sequence-based features are independent of pH and temperature.

### Machine learning

Here we used the Random Forest binary classifier using the Weka toolkit^57^ to train our predictive models. Random Forest is an ensemble-learning robust classification algorithm, in which multiple decision trees are included over a random subset of features and decide the output via majority voting. The model was trained using 10-fold cross-validation and performance evaluated by area under the Receiver Operating Characteristic (AUROC) curve, precision and accuracy. Further validation of the models was performed using a blind-test set of 184 mutations, which were non-redundant at the position-level with mutations in the training set. Analysis of the final model revealed a set of structural features that distinguished between susceptible and resistant pncA point mutations.

### Webserver development

The server front-end was built using materialize CSS framework version 1.0.0, while the backend was built in Python via the Flask framework (version 0.12.2). It is hosted on a Linux server running Apache.

### Sequencing and DST of clinical isolates

Genomic DNA was extracted according to the mechanical cell disruption and ethanol precipitation method outlined in Votintseva 2015^58^ with slight modifications. Briefly, no pre-treatment was used and approximately 3 × 1 µL loops of culture were dispersed in 700 µL TE buffer (Sigma Aldrich) as the starting material. The precipitated DNA pellet was only washed once and resuspended into 50 µL EB Buffer (Qiagen) at 55 °C for 10 minutes with regular vortexing. Finally, samples were centrifuged 3 min at 13,000 rpm and 45 µL of DNA extract was transferred into a clean tube for downstream processing. Each extract was interrogated for *Mycobacterium tuberculosis* viability by inoculating 15 µL of DNA extract into MGIT tube (Becton Dickinson, UK) and incubated in the Bactec MGIT 960 system (Becton Dickinson, UK). Unique dual indexed libraries were prepared using the Nextera XT DNA sample preparation kit (Illumina). Libraries were sequenced on the Illumina NextSeq. 500 with 150-cycle paired end chemistry as described by the manufacturer’s protocols.

Sequences were aligned to H37Rv (NC_0009623.3) and small nucleotide variations (SNV) mutations in *pncA* were identified using LoFreq (http://csb5.github.io/lofreq/). SNVs with a frequency > 0.6 were used to compare the genotype of isolates to the phenotype observed using standard laboratory methods for PZA susceptibility testing.

## Supplementary information

Supplementary Information .
