# Supplementary material for: Structure guided prediction of Pyrazinamide resistance mutations in pncA
Source: Sci Rep. 2020 Feb 5;10:1875. doi: 10.1038/s41598-020-58635-x (PMC7002382; doi:10.1038/s41598-020-58635-x)
Supplement: Supplementary file 1 — Supplementary Information . [file 41598_2020_58635_MOESM1_ESM.docx]

**Supplementary Materials**

**Structure guided prediction of Pyrazinamide resistance mutations in pncA**

Malancha Karmakar^1,2,3^, Carlos H. M. Rodrigues^2,3^, Kristy Horan^4^, Justin T. Denholm^1^ , David B. Ascher^2,3,5^

^1^Victorian Tuberculosis Program, Melbourne Health and Department of Microbiology and Immunology, University of Melbourne, Melbourne, Victoria, Australia

^2^Structural Biology and Bioinformatics, Baker Heart and Diabetes Institute, Melbourne, Victoria, Australia

^3^Department of Biochemistry and Molecular Biology, Bio21 Institute, University of Melbourne, Melbourne, Victoria, Australia

^4^Microbiological Diagnostic Unit Public Health Laboratory, University of Melbourne at The Peter Doherty Institute for Infection &Immunity, Melbourne, Victoria, Australia

^5^Department of Biochemistry, University of Cambridge, CB2 1GA, UK

**Table S1: The list of different features used to analyze and build the empirical model for predicting novel resistance mutations in PZA.**

| **Parameters** | **Effect measured** | **Technique** | **Mean of R mutations** | **Mean of S mutations** | **Mean** | **p-value*** | **95% CI** |
| --- | --- | --- | --- | --- | --- | --- | --- |
| DUET  (Kcal/mol) | Protein Stability | Graph-based signatures | -1.13 | -0.57 | -0.85 | 5.28 e^-14^ | [-0.77 to -0.92] |
| Distance from Ligand (Å) | Distance of the mutation from the drug (PZA) binding site | Perl script (in-house) | 9.48 | 13.03 | 11.25 | 1.41 e^-15^ | [10.81 to 11.65] |
| DynaMut  (Kcal/mol) | Conformational flexibility | Normal mode analysis | -0.24 | 0.06 | -0.08 | 6.55 e^-06^ | [-0.02 to -0.16] |
| mCSM-Stability  (Kcal/mol) | Protein Stability | Graph-based signatures | -1.10 | -0.64 | -0.87 | 3.83 e^-12^ | [-0.80 to -0.93] |
| RSA (Å) | Environmental characteristics | Python script (in-house) | 0.18 | 0.39 | 0.28 | < 2.2 e^-16^ | [0.27 to 0.31] |
| SNAP | Functional effect of single nucleotide substitution | Neural Networks | 49.83 | 4.81 | 27.73 | < 2.2 e^-16^ | [23.25 to 31.39] |
| Ligand binding affinity (mCSM-Lig) | Ligand binding affinity | Graph-based signatures | -0.98 | -0.84 | -0.90 | 0.14 | [-0.82 to -0.99] |
| PROVEAN | Functional effect of single nucleotide substitution | alignment-based score approach | -5.42 | -3.04 | -4.23 | < 2.2 e^-16^ | [-4.01 to -4.45] |
| SDM  (Kcal/mol) | Protein Stability | Graph-based signatures | -1.15 | -0.30 | -0.72 | 1.14 e^-07^ | [-0.57 to -0.88] |
| Dihedral angle (Phi) | Environmental characteristics | Python script (in-house) | -73.18 | -71.36 | -71.71 | 0.58 | [-67.30 to -77.22] |
| Dihedral angle (Psi) | Environmental characteristics | Python script (in-house) | 50.65 | 36.98 | 44.35 | 0.11 | [36.23 to 51.41] |
| Residue Depth | Environmental characteristics | Python script (in-house) | 1.09 | 0.74 | 0.92 | < 2.2 e^-16^ | [0.89 to 0.95] |
| ENCoM | Conformational flexibility | Normal mode analysis | 0.09 | 0.09 | 0.09 | 0.87 | [0.06 to 0.13] |
| Relative  b-factor | Environmental characteristics | Python script (in-house) | 3.03 | 3.18 | 3.10 | 2.57 e^-10^ | [3.09 to 3.13] |

*p-value calculated using Welch two-sample t-test

**Table S2: List of performances for predictive models trained on individual classes of attributes and all attributes combined using 10-fold cross validation.**

| **Attributes** | **Class label** | **Accuracy** | **MCC** | **Precision** | **Recall** | **F-measure** | **ROC AUC** |
| --- | --- | --- | --- | --- | --- | --- | --- |
| Stability | R | 0.57 | 0.21 | 0.61 | 0.57 | 0.59 | 0.62 |
|  | S | 0.64 | 0.21 | 0.59 | 0.64 | 0.62 | 0.62 |
|  | Weighted Avg. | 0.61 | 0.21 | 0.60 | 0.60 | 0.60 | 0.62 |
| Dynamics | R | 0.55 | 0.14 | 0.57 | 0.55 | 0.56 | 0.62 |
|  | S | 0.58 | 0.14 | 0.57 | 0.58 | 0.57 | 0.62 |
|  | Weighted Avg. | 0.56 | 0.14 | 0.57 | 0.57 | 0.57 | 0.62 |
| Evolutionary Conservation | R | 0.66 | 0.32 | 0.66 | 0.66 | 0.66 | 0.70 |
|  | S | 0.65 | 0.32 | 0.66 | 0.65 | 0.66 | 0.70 |
|  | Weighted Avg. | 0.65 | 0.32 | 0.66 | 0.66 | 0.66 | 0.70 |
| Ligand interactions | R | 0.62 | 0.26 | 0.63 | 0.62 | 0.62 | 0.68 |
|  | S | 0.64 | 0.26 | 0.62 | 0.64 | 0.63 | 0.68 |
|  | Weighted Avg. | 0.63 | 0.26 | 0.63 | 0.63 | 0.63 | 0.68 |
| Backbone geometry (Structural environment) | R | 0.61 | 0.27 | 0.64 | 0.62 | 0.63 | 0.70 |
|  | S | 0.64 | 0.27 | 0.63 | 0.64 | 0.64 | 0.70 |
|  | Weighted Avg. | 0.63 | 0.27 | 0.63 | 0.63 | 0.63 | 0.70 |
| **Predictive Model** | **R** | **0.75** | **0.60** | **0.84** | **0.75** | **0.79** | **0.83** |
|  | **S** | **0.85** | **0.60** | **0.77** | **0.85** | **0.81** | **0.83** |
|  | **Weighted Avg.** | **0.80** | **0.60** | **0.80** | **0.80** | **0.80** | **0.83** |


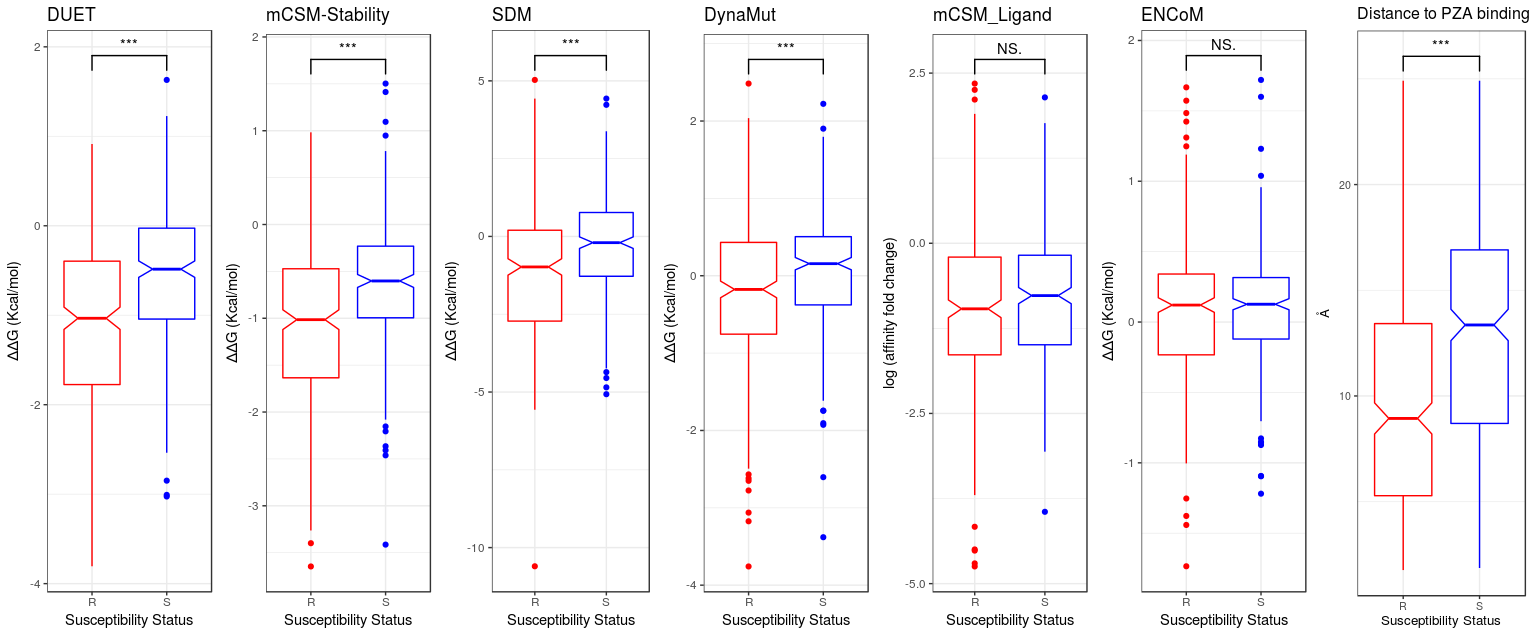


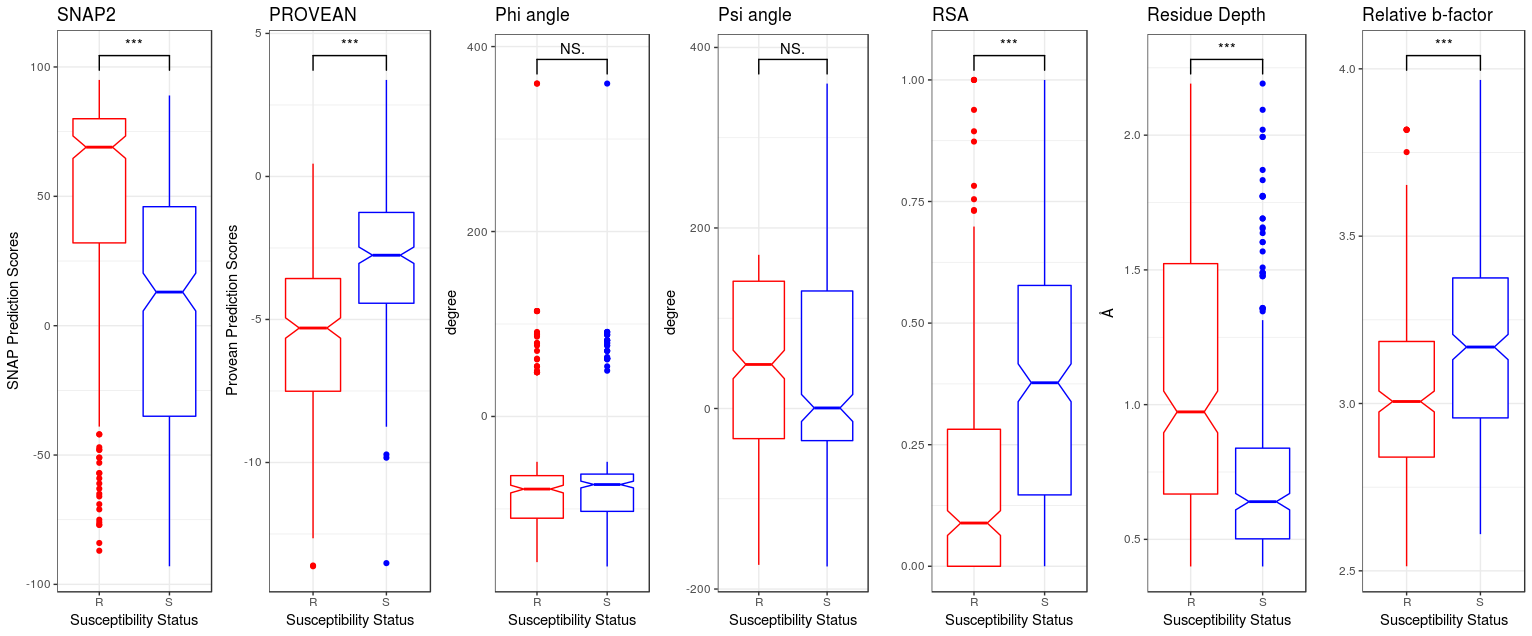


**Figure S2: Boxplots comparing features calculated for resistant and susceptible variants.** The resistant associated mutations (R) are represented as red and the susceptible mutations (S) as blue. NS- non-significant; *** p < 0.0001 by Welch two sample t-test.

**
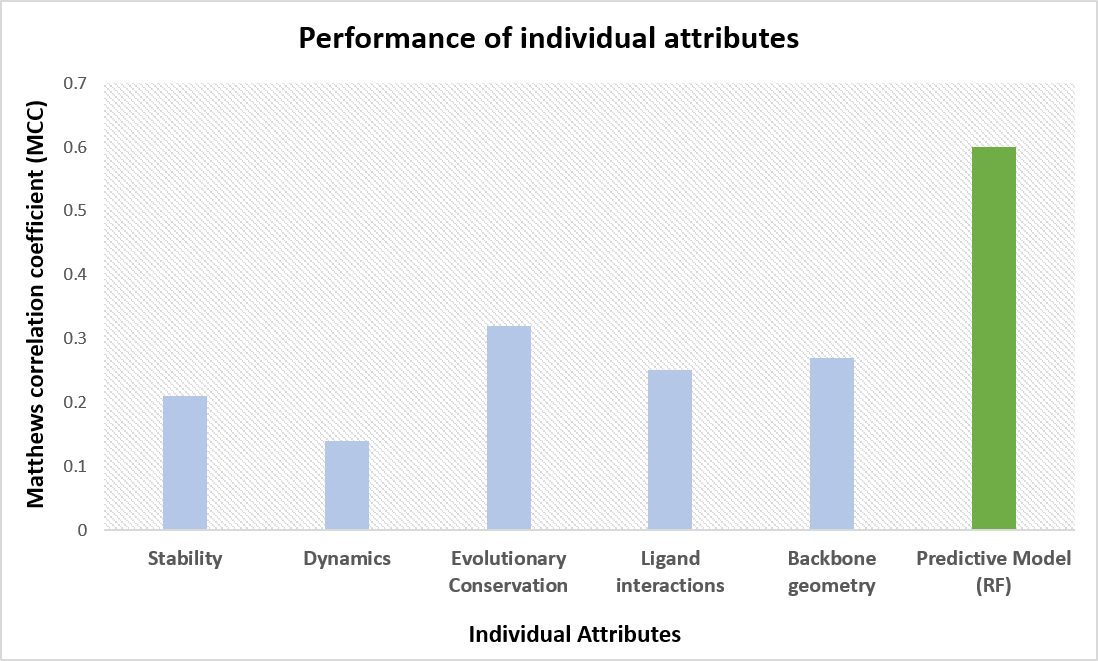
**

**Figure S1: Performance of predictive model trained on single class of features.** The Random Forest algorithm was trained using 10-fold cross validations using each single class of features (first five bars from left to right; blue bars) and with the combination of all features (green bar). We observe the predicted MCC score is low when we use only a single class of feature for training. However, a significant increase is observed when different features are combined for the predictive model.


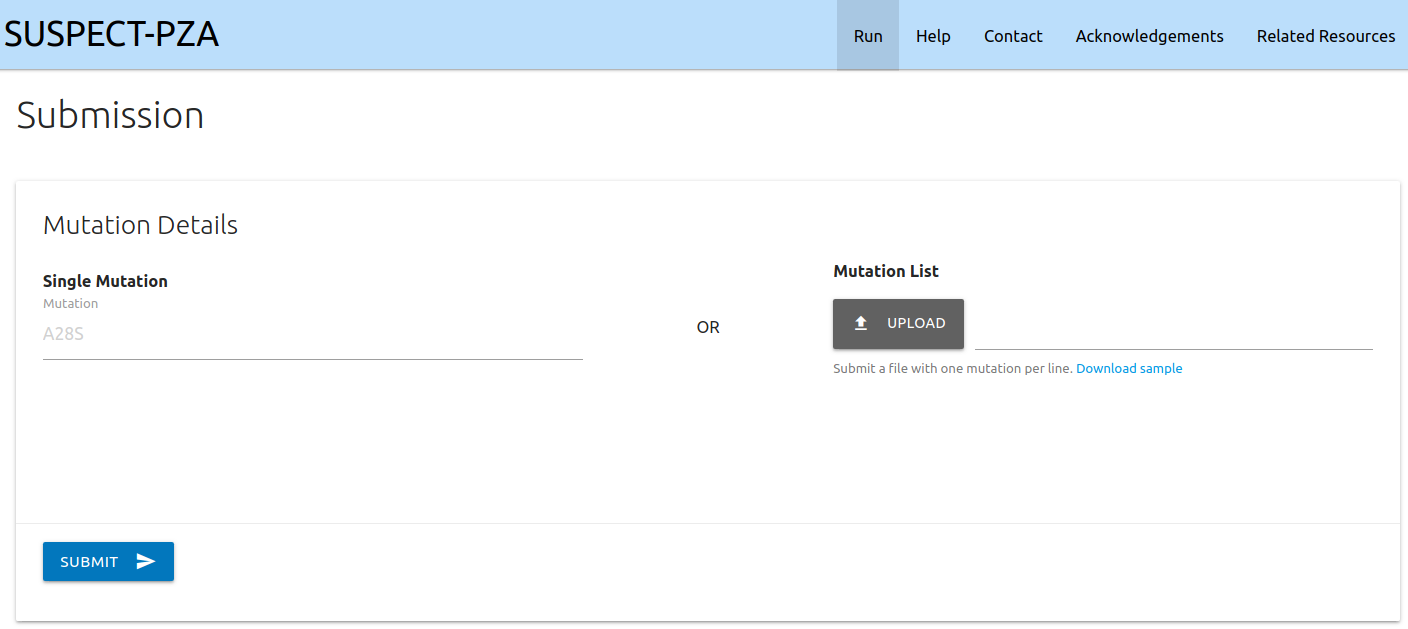


**Figure S3: SUSPECT-PZA submission page.** The submission page for single point mutation or to upload a list of single point mutations. This can be accessed via the menu item Run on the top bar.


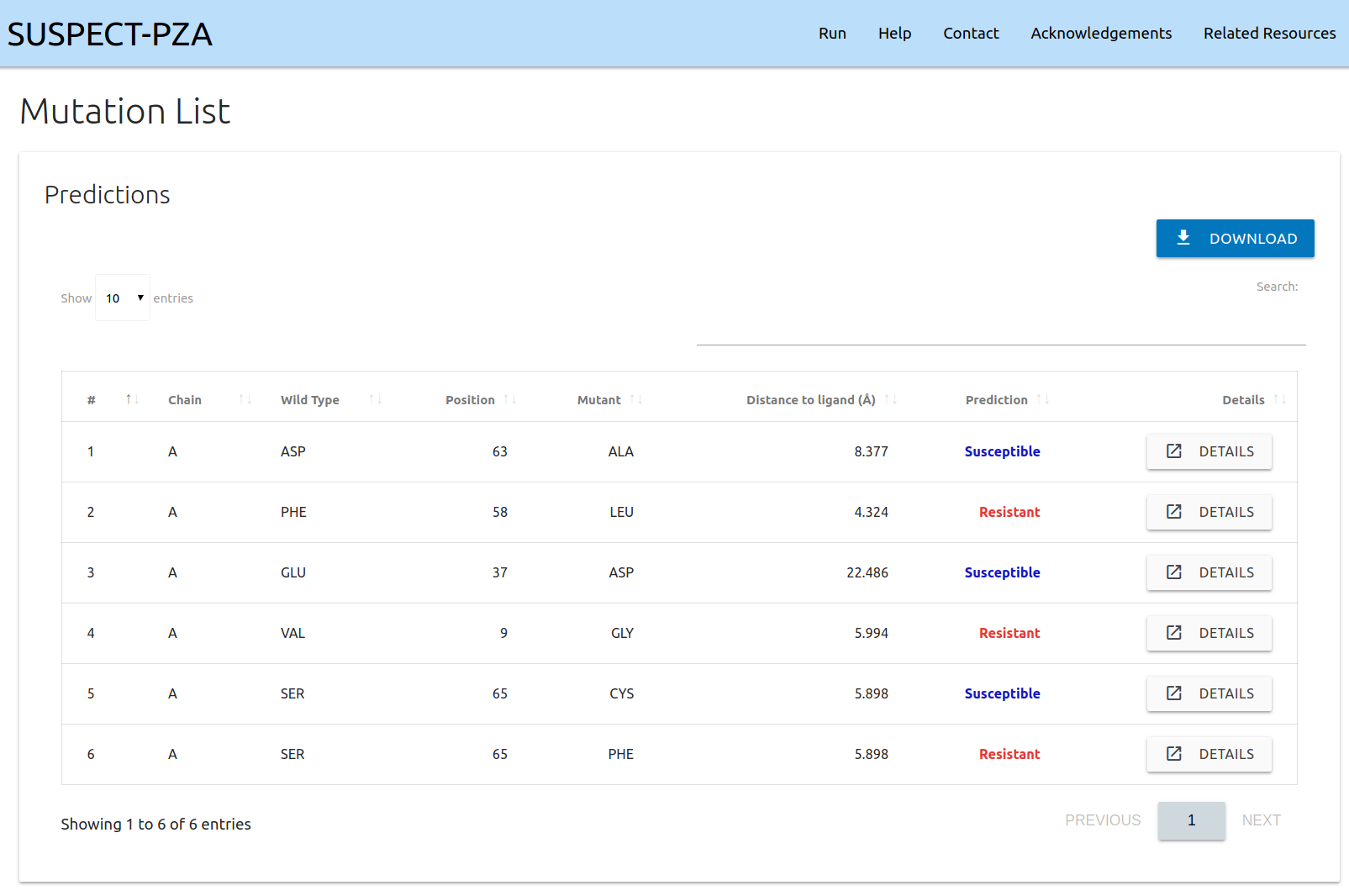


**Figure S4: SUSPECT-PZA results page for a list of single point mutations.** The predictions for every single point mutation will be displayed as a table in the order of input as in the mutation list. The results can be downloaded as a .csv file by clicking on the Download button on the top right corner. All the analysis discussed for the single mutation option can be analysed for each single mutation on the table through the Details button of each row.


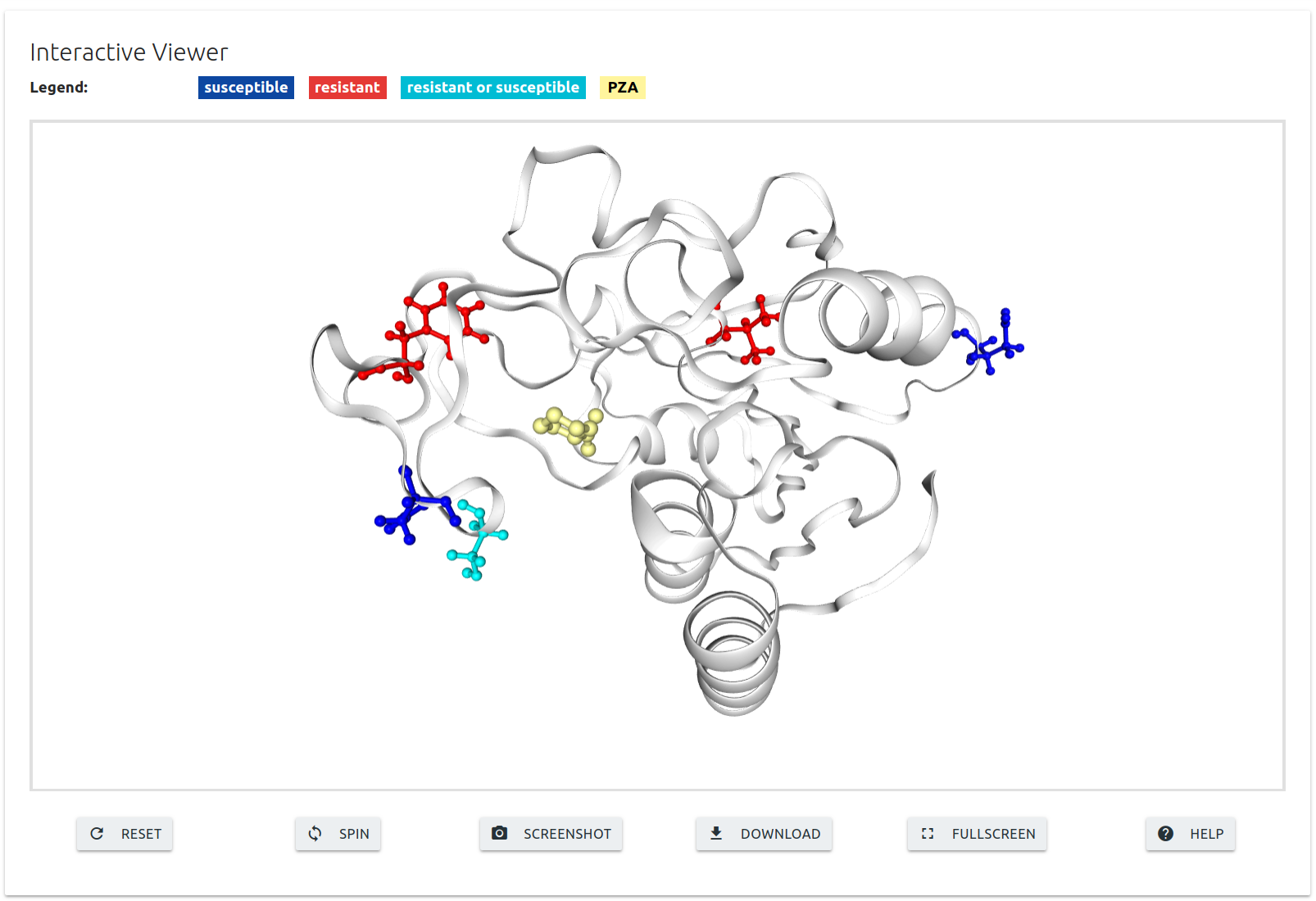


**Figure S5:** **SUSPECT-PZA interactive viewer showing the mutations on the secondary structure.** Result page displaying the location of the susceptible (blue, ball and stick representation) and resistant (red, ball and stick representation) mutations. For the amino acid position which harbors both susceptible and resistant mutation is shown in cyan (ball and stick representation).
